# Analysis of compound health impacts of heatwave and COVID-19 in Korea from May to September in 2020

**DOI:** 10.1038/s41598-023-41880-1

**Published:** 2023-09-09

**Authors:** Haemin Park, Sang-Min Lee, Woo Joong Kim, Yeora Chae

**Affiliations:** Korea Environment Institute, 370 Sicheong-Daero, Sejong, 30147 Republic of Korea

**Keywords:** Environmental impact, Risk factors

## Abstract

The number of non-accidental deaths and heat-related illnesses due to the co-occurrence of heatwaves and COVID-19 has been identified to estimate compound health impacts between two risks. We have analyzed data from historical years (2013–2019) to calculate the baseline values of the number of non-accidental deaths and heat-related illness patients from May to September using a quasi-Poisson generalized linear model and compared them to data from 2020 in Korea. We also assessed the relative risk and absolute cumulative number of non-accidental deaths and heat-related illnesses in the summer of 2020 in Seoul, Daegu, and Gyeongnam region of Korea. In the Summer of 2020, Korea experienced 0.8% of non-accidental excess deaths, with the highest in August, and 46% of reduction was observed in heat-related throughout the study period, except in Daegu, where excess of heat-related illness occurred in August. The relative risk (RR) of non-accidental deaths at 33.1 °C, was 1.00 (CI 0.99–1.01) and 1.04 (CI 1.02–1.07) in 2013–2019 and 2020, respectively. The RR of heat-related illness at 33.1 °C, was 1.44 (CI 1.42–1.45) and 1.59 (CI 1.54–1.64) in 2013–2019 and 2020, respectively. The absolute cumulative trends of non-accidental deaths and heat-related illnesses were similar in the three regions, indicating increased non-accidental deaths and decreased heat-related illnesses at similar temperatures in 2020. During the COVID-19 pandemic, the fear of infection by the virus and the limited access to healthcare services led to changes in health-seeking behaviors. These results indicate social distancing could have had adverse impacts on other health conditions. A comprehensive health risk assessment is important when facing simultaneous risks, such as heatwaves and pandemics, in the implementation of effective countermeasures.

## Introduction

Modern society faces many risks. Public health emergencies due to COVID-19 were started from January 2020. Furthermore, the adverse health effects of climate change have been increasing continuously in many parts of the world. In England, the highest daily temperature in 2020 was recorded at the end of July^1^. In 2021, the Pacific Northwest areas of the US and Canada experienced the greatest heatwave in June^2^. In 2022, an unprecedented heatwave occurred in East Asia and Eastern China^3,4^. Moreover, the crisis of infectious diseases that we have encountered in recent years is aggravated by climate change^5^. Such risks could be interconnected, including the risks from the transmission of vector-borne diseases, heatwaves, wildfires, floods, and food security, which could disproportionately affect vulnerable population^6^.

Heatwaves have various impacts on our lives, including a severe impact on our health. In 2018, Japan experienced extreme heatwaves starting in the middle of July, and unprecedented admissions for heat-related illnesses were reported^7^. A study from China revealed that heatwaves could affect non-accidental deaths, cardiovascular diseases, stroke, respiratory diseases, and circulatory diseases^8^. In Korea, an increasing trend in the daily maximum temperature and the number of heatwaves occurring per day has been reported^9^. In 2018, in Korea, the highest maximum temperature was observed, reporting 41 °C of maximum temperature in the Hongcheon area (August 1st, 2018), while the highest regional temperature was observed in Seoul, Chuncheon, Suwon, and Daejeon. In Korea, during the summer of 2018, an average of 31.15 days of heatwaves occurred, with 44,094 and 145 heatwave-related illnesses and deaths, respectively^10^.

As of January 15, 2023, 17,795,357 cumulative confirmed cases of COVID-19 have been reported in Korea. Furthermore, the period from January 2020 to January 2022 has been divided into four phases reflecting the temporal and regional characteristics of COVID-19^11^: Phase 1 (from January 20 to August 11, 2020), which includes the first wave epicentering in Daegu and Gyeongbuk area during early February-May in 2020; Phase 2 (from August 12 to November 12, 2020), which includes the second wave mainly occurring in the capital area during the middle of August-middle of November in 2020; Phase 3 (from November 13, 2020, to July 21, 2021), which includes the third waves in winter during the middle of November-end of January; and Phase 4, which includes the fourth wave that started in early July of 2021. To mitigate the spread of confirmed COVID-19 cases, non-pharmaceutical measures were implemented, including vaccination, testing, tracing, quarantining, and social distancing^12^, leading to changes in the economic sector workforce and an increase in unemployment^13^, implying that COVID-19 could function as another risk source in people’s lives.

Moreover, the risks do not act alone; multiple risks, such as heatwaves, droughts, pandemics, and energy crises, often have more severe impacts on society. Furthermore, risks can be transferred to and/or amplified in other sectors and countries. Climate change and the COVID-19 pandemic could be interconnected, and their coincidence could generate new risks. Globally, populations at risk of climate exposure experiencing the severe impacts of COVID-19^14,15^ could aggravate their existing vulnerability to health, social, and environmental risks. Moreover, individuals with pre-existing comorbidities such as hypertension, cardiovascular diseases, diabetes mellitus, and chronic kidney disease are known to be severely affected by COVID-19^16,17^ and are susceptible to extreme climate events, including heatwaves^18^. Furthermore, older people with multi-chronic conditions, low income, and living alone are at risk due to impaired physiological mechanisms and the low accessibility of healthcare facilities during heatwaves^19–22^.

Although the co-occurrence of COVID-19 and heatwaves is known to aggravate risks, only a few studies have focused on the health impacts of COVID-19 during heatwaves^23^. This study aimed to determine how social distancing and accessibility to medical services affect heatwave-related health risks. We analyzed heatwave-related health impacts during the summer of 2020 and the COVID-19 period compared to 2013–2019. Furthermore, we compared the number of non-accidental deaths and patients with heat-related illnesses in Korea with and without COVID-19 during the summer.

## Methods

### Data

#### Statement on guidelines

This study complied with relevant guidelines and regulations. Datasets on heat-related illnesses and cause-specific mortality are publicly available and do not include any identifiable information. As there was no direct involvement by the participants, patient consent and ethical approval were not required for this study.

#### Mortality and heat-related illness data

Data on daily non-accidental deaths from 2013 to 2020 were retrieved from the MicroData Integrated Service (MDIS) website^24^, which provides cause-specific mortality data by region, sex, and age. Non-accidental deaths were defined as deaths from all-causes deaths, except accidental deaths corresponding to code V01-Y98 according to the International Statistical Classification of Diseases 10th Revision (ICD-10). Non-accidental deaths were aggregated from 17 administrative regions in Korea, and daily mortality data including the summer season (May-September) of 2013–2020 were used. The daily number of heat-related illness patients during the study period was obtained through the National Health Insurance Sharing Service (NHIS) customized DB^25^, focusing on outpatients and inpatients with heat-related illnesses who were coded as T67 by ICD-10. Regional population data were downloaded from the website operated by the Ministry of the Interior and Safety in Korea and combined with data on daily mortality and heat-related illness among patients.

#### Temperature and the number of days that heatwave occurred

The regionally averaged daily maximum temperature data were obtained by averaging the 1 km of spatially gridded data into 17 administrative regions^26^. These data were interpolated from adjacent 5 km spatial temperature data, and other topographic (elevation of terrain, distance from coast, and aspect ratio) and surface (impermeable area, vegetation area, and density of buildings) variables were used to reflect regional characteristics during the interpolation. The average regional daily maximum temperature in Korea was used to calculate the daily maximum temperature in South Korea. The maximum temperatures in Korea were retrieved from the Korea Meteorological Administration Weather Data Service (https://data.kma.go.kr/resources/html/en/aowdp.html) and used for validation. The number of heatwaves, defined as maximum temperatures exceeding 33 °C, was obtained from the Korea Meteorological Administration Weather Data Service.

### Analysis

#### Baseline deaths

To estimate the changes in deaths and heat-related illnesses during the summer season (May–September) in 2020, the baseline non-accidental deaths were obtained using the summer season from 2013 to 2019. To reflect the recent trend of mortality in Korea, we retrieved data for eight years (from 2013 to 2020) and used mortality data from 2013 to 2019 to predict the historical baseline. A predicted baseline of daily non-accidental deaths was constructed for each of the 17 administrative regions in Korea using a quasi-Poisson generalized linear model.

We regressed non-accidental deaths and heat-related illnesses (outcome variables) on date (*d*) and region (*r*) using the model Eqs. (1) and (2):

1 $$Outcome\, variables_{ d,r} \sim Poisson\left( {\lambda_{d,r} } \right)$$

2 $$log\left( {\lambda_{d,r} } \right) = \beta_{0} + \beta_{1} *trend_{d,r} + \beta_{2} *dow_{d,r} + \beta_{3} *Tmax_{d,r} + offset\left( {log\left( {population_{d,r} } \right)} \right)$$

where *trend*_*d,r*_ is a continuous variable from the start date to the end date of the study period as the trend at date *d* and region *r*; dow_*d,r*_ is the day of the week at date *d* and region *r,* Tmax_*d,r*_ is the regionally averaged daily maximum temperature at date *d* and region *r.* The natural logarithm of the *population*_*d,r*_ was used as an offset. The parameters ${\beta }_{0}$, ${\beta }_{1}$, ${\beta }_{2}$, and ${\beta }_{3}$ are coefficients to be estimated for each potential confounder. We used the quasilikelihood considering the overdispersion, assuming that the variance is Var(*Outcome variables*_*d,r*_) = *ψ* ${\lambda }_{d,r.}$ Studies considering temperature on mortality outcomes vary in their assumptions of linearity, non-linearity using a spline function, and a distributed lag non-linear model^27–29^. As the purpose of this study was to predict health-related outcome variables during the summer season, we assumed a linear relationship between temperature and outcome variables.

The predicted values of non-accidental deaths and heat-related illnesses in 2020 were compared with the observed values. Two indices, absolute changes (observed-predicted) and percent changes ((observed-predicted)/predicted × 100), were calculated to quantify the difference in mortality and disease occurrence during the COVID-19 period.

#### Model validation

Model validation was conducted using temperature variables, including the average spatially gridded maximum temperature, population-weighted spatially gridded maximum temperature, and maximum temperature from other data sources from the Korea Meteorological Administration (KMA). The model prediction was validated using a dataset from 2013 to 2019. The number of non-accidental deaths and heat-related illness data in 2019 were used as the test dataset by comparing baseline data obtained by using data from 2013 to 2018. To estimate the error in the prediction model, projection errors based on the mean percentage error (MPE) and root mean square error (RMSE) were used as indices for validation (Supplementary Table S1).

#### Relative risk and absolute cumulative function

To evaluate the relative risk of non-accidental deaths and heat-related illnesses in the summer of 2020, a general additive model (GAM) was used to control for potential confounders, such as the day of the week and trend, using the following equation:

3 $$\ln \left[ {{\text{E}}\left( {\text{Y}} \right)} \right] = \beta_{0} + \beta_{1} *trend + \beta_{2} *\left( {dow} \right) + s\left( {T_{max} } \right)$$

where E(Y) refers to non-accidental deaths and heat-related illnesses in Korea, *s* denotes the smooth functions for the ${T}_{max}$ (daily maximum temperature), and *trend* and *dow* account for the effect of long-term trends and day of the week. The relative risks (RRs) for 2020 and 2013–2019 were calculated and compared. If the RR is greater than 1, the event is more likely to occur if there is exposure. Therefore, we compared the maximum temperature at the point where RR was greater than 1.

The absolute cumulative function of non-accidental mortality and heat-related illness occurrences (F_i_) at maximum temperature was calculated for each year (y) from 2013 to 2020 using the following equation:

4 $${\text{F}}_{{({\text{y}},{\text{ t}})}} = {\text{ f}}_{{({\text{y}},{ 1})}} + {\text{f}}_{{({\text{y}},{ 2})}} + {\text{f}}_{{({\text{y}},{ 3})}} + \cdots {\text{F}}_{{({\text{y}},{\text{ t}})}}$$

where t refers to the maximum temperature rearranged from the lowest to the highest, and F_(y, t)_ refers to the non-accidental mortality and heat-related illness occurrence per 100,000 people at the maximum temperature. Similar to previous studies^30^, the absolute cumulative function could provide an intuitive understanding of the statistical distribution, allowing us to observe how non-accidental mortality and heat-related illness occurrences were different in 2020 compared to historical years.

## Results

**Figure 1:**
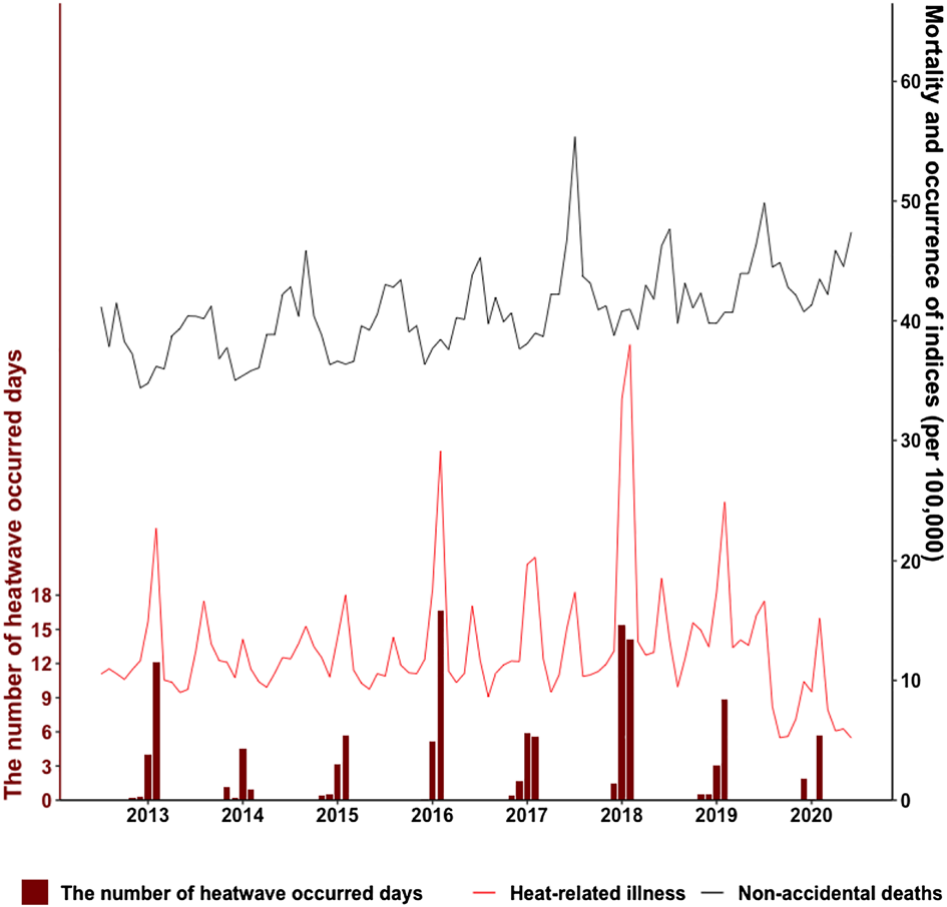
The number of heatwave-occurring days during the summer (May–September), the monthly mortality rate of non-accidental deaths, and the occurrence of heat-related illness per 100,000 people during 2013–2019 in Korea.

Figure 1 shows the number of heatwave-occurring days, the mortality rate of non-accidental deaths, and the occurrence of heat-related illness and the regional characteristics of these values, including average maximum temperatures are shown in Supplementary Fig. S1 and the annual trend is shown in Supplementary Tables S2–S6. From 2013 to 2020, the patterns of the number of heatwave-occurring days varied by year; Korea experienced the most severe heatwave in 2018, recording a total of 31 heatwave-occurring days, with the highest occurrence (108.5 heat-related illness patients per 100,000 people) during the summer. In 2020, 7.7 heatwave-occurring days were reported, which was similar to the 2014 record of 6.7 heatwave-occurring days. In total, 48.4 heat-related illness patients per 100,000 people occurred in 2020, and the number of patients decreased compared to that in 2014 (56 heat-related illness patients per 100,000 people). Moreover, non-accidental deaths were the highest in 2020, with 209.9 deaths per 100,000 people.

**Figure 2:**
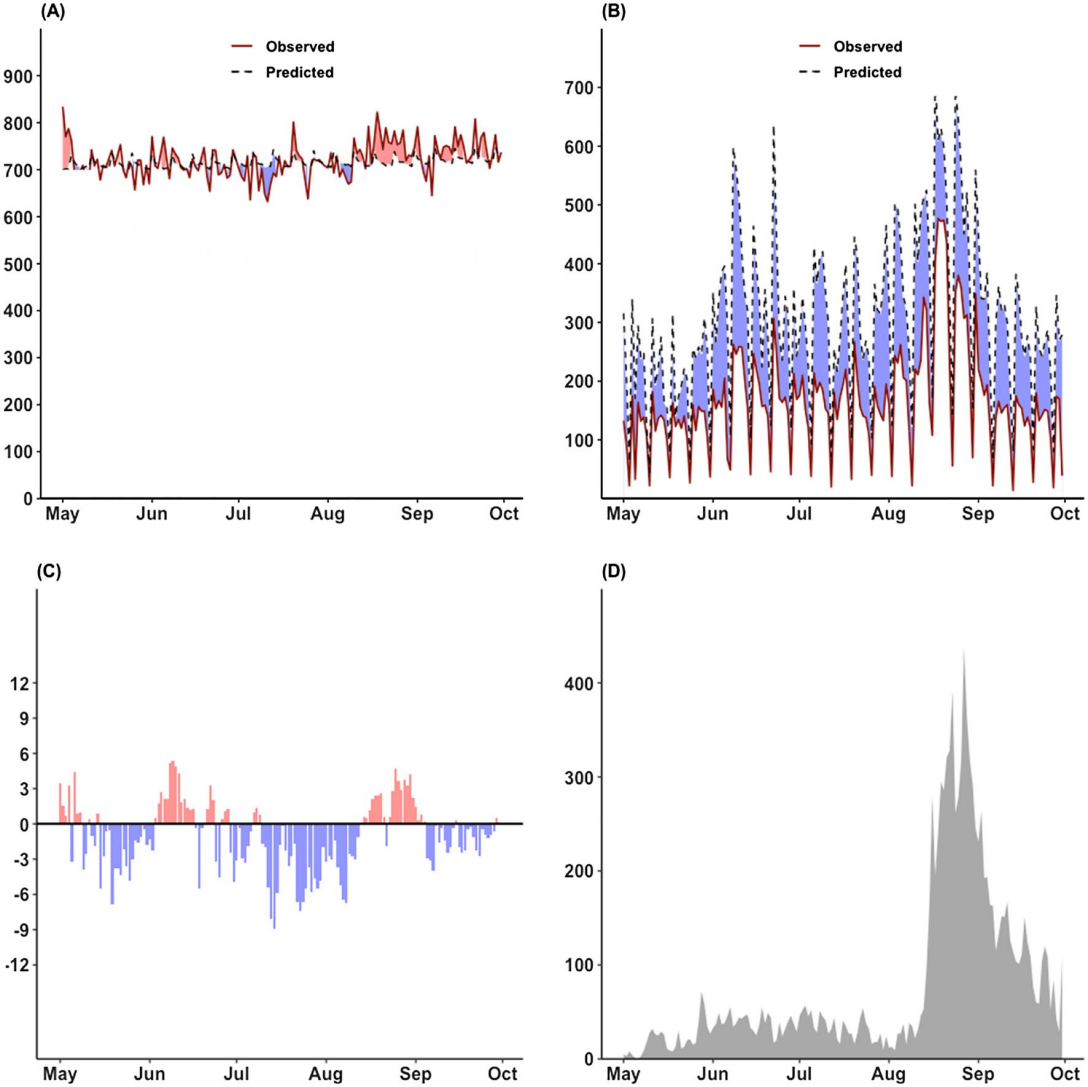
The results of (**A**) the observed and predicted values of non-accidental deaths; (**B**) the observed and predicted values of heat-related illness; (**C**) the maximum temperature anomaly compared to the average value of the previous year (2013–2019); and (**D**) the number of COVID-19 confirmed cases during May–September 2020 in Korea.

In 2020, throughout the study period of May–September, 110,377 non-accidental deaths were reported in Korea, exceeding the 892 predicted deaths (Fig. 2). Excess non-accidental deaths were the highest in August, with an additional 658 deaths; 4% of excess deaths were from COVID-19 (n = 26), while 96% of excess deaths were due to other causes of death. In 2020, 46% of deficit in the patients with heat-related illnesses were observed throughout the study period, with the highest 63% of deficit in September. Moreover, a positive maximum temperature anomaly was observed in June and August, and Korea experienced a second wave of COVID-19 with an epicenter in the capital metropolitan area in mid-August.

The magnitude of excess deaths differed by region in Korea. Tables 1 and 2 show the results of the excess changes in non-accidental deaths and heat-related illness percentages. Supplementary Table S7 shows the characteristics of maximum temperature and COVID-19 confirmed cases by region in 2020. In Korea, 3% of non-significant non-accidental deaths were observed in August, with significant excess non-accidental deaths in Busan (5.1%), Gwangju (7.7%), Daejeon (5.5%), Chungnam (6.8%), Gyeongbuk (7.7%), and Gyeongnam (7.1%). Seoul, the epicenter of the second wave in Korea, experienced 0.8% of non-significant excess non-accidental deaths. Moreover, in Daegu (the epicenter of the first wave), the highest cumulative COVID-19 confirmed cases occurred until the summer of 2020, and exposure to the highest maximum temperature resulted in 3.7% of non-significant excess non-accidental deaths in August. Gyeongnam, which experienced the lowest COVID-19 confirmed cases in Korea during the summer, experienced non-significant but constant excess non-accidental deaths throughout the summer, with 7.1% of the highest significant excesses in August.

**Table 1:** The results of percent excess changes in non-accidental deaths in Korea during the summer of 2020.

	May	June	July	August	September
Korea	0.6 (− 2.6 to 4)	0.3 (− 2.7 to 3.5)	− 2 (− 5 to 1.1)	3 (− 0.2 to 6.4)	2.2 (− 1 to 5.7)
Seoul	1.4 (− 1 to 3.8)	− 0.7 (− 2.8 to 1.6)	− 0.8 (− 3 to 1.4)	0.8 (− 1.4 to 3.2)	4.1 (1.7 to 6.5)
Busan	0.1 (− 3 to 3.3)	0.8 (− 2 to 3.9)	− 1.3 (− 4.3 to 1.7)	5.1 (1.9 to 8.6)	2.1 (− 1 to 5.4)
Daegu	− 3 (− 6.5 to 0.8)	− 4.4 (− 7.8 to − 0.8)	0.1 (− 3.6 to 4)	3.7 (− 0.2 to 7.9)	1 (− 2.9 to 5.1)
Incheon	0.7 (− 3.1 to 4.8)	3.1 (− 0.4 to 6.9)	− 2 (− 5.4 to 1.6)	3.4 (− 0.3 to 7.3)	0.7 (− 3 to 4.6)
Gwangju	− 3 (− 7.6 to 2.2)	0.8 (− 3.8 to 5.8)	− 1.1 (− 5.7 to 4)	7.7 (2.6 to 13.3)	− 0.4 (− 5.2 to 4.9)
Daejeon	− 1 (− 5.9 to 4.5)	− 2 (− 6.6 to 3.2)	4.3 (− 0.7 to 9.9)	5.5 (0.3 to 11.3)	2.8 (− 2.4 to 8.5)
Ulsan	11.5 (5.2 to 18.6)	1 (− 4.4 to 7.1)	4.1 (− 1.7 to 10.6)	1.3 (− 4.6 to 7.9)	9.3 (3 to 16.4)
Sejong	13.1 (− 0.7 to 31.4)	13.5 (0.1 to 30.8)	39.2 (22.7 to 60.9)	9.8 (− 3.5 to 27.5)	42.9 (25.1 to 66.5)
Gyeonggi	− 0.4 (− 2.4 to 1.6)	0.9 (− 1 to 2.8)	− 4.9 (− 6.6 to − 3.1)	0.5 (− 1.4 to 2.4)	− 0.1 (− 2 to 1.9)
Gangwon	3.1 (− 1.1 to 7.7)	− 0.3 (− 4.2 to 3.8)	− 5.4 (− 9.1 to − 1.3)	3.4 (− 0.7 to 7.9)	1 (− 3.2 to 5.6)
Chungbuk	3.9 (− 0.2 to 8.5)	6.8 (2.8 to 11.2)	4.4 (0.4 to 8.8)	− 0.5 (− 4.4 to 3.7)	2.1 (− 2.1 to 6.6)
Chungnam	− 4.3 (− 7.8 to − 0.5)	− 2.9 (− 6.2 to 0.7)	0 (− 3.5 to 3.6)	6.8 (3.1 to 10.9)	− 0.6 (− 4.2 to 3.2)
Jeonbuk	− 1 (− 4.7 to 3)	1.1 (− 2.4 to 5)	− 5.8 (− 9.2 to − 2.1)	2.6 (− 1.2 to 6.6)	3.2 (− 0.6 to 7.4)
Jeonnam	3.9 (0.2 to 7.9)	− 1 (− 4.4 to 2.5)	− 4.6 (− 7.9 to − 1.1)	− 0.9 (− 4.3 to 2.8)	5 (1.3 to 9.1)
Gyeongbuk	4.7 (1.6 to 7.9)	3.4 (0.5 to 6.5)	− 5.3 (− 8.1 to − 2.4)	7.7 (4.6 to 11)	2.5 (− 0.6 to 5.7)
Gyeongnam	1.2 (− 1.9 to 4.5)	0.2 (− 2.7 to 3.3)	2.2 (− 0.8 to 5.5)	7.1 (3.8 to 10.5)	3.1 (− 0.1 to 6.6)
Jeju	− 13.6 (− 19.6 to − 6.6)	− 12.4 (− 18.1 to − 5.8)	− 13.1 (− 18.9 to − 6.4)	− 1.7 (− 8.5 to 6.2)	6 (− 1.3 to 14.6)

**Table 2:** The results of percent excess changes in heat-related illness patients in Korea during the summer of 2020.

	May	June	July	August	September
Korea	− 44.5 (− 49.2 to − 38.9)	− 48.7 (− 52.8 to − 43.9)	− 48.6 (− 52.8 to − 43.6)	− 40.7 (− 45.7 to − 34.8)	− 51 (− 55.2 to − 46)
Seoul	− 56 (− 58.9 to − 52.7)	− 58.3 (− 60.8 to − 55.3)	− 58.6 (− 61.2 to − 55.7)	− 59.2 (− 61.8 to − 56.3)	− 63.3 (− 65.7 to − 60.5)
Busan	− 54.8 (− 58.3 to − 50.6)	− 51.5 (− 55 to − 47.5)	− 48 (− 51.9 to − 43.4)	− 28.1 (− 33.5 to − 21.6)	− 44.1 (− 48.4 to − 39.1)
Daegu	− 21.8 (− 31.1 to − 9.8)	− 30.7 (− 38.6 to − 20.4)	− 23.9 (− 33 to − 12)	35.9 (19.7 to 57.2)	4 (− 8.9 to 21)
Incheon	− 32.4 (− 39.3 to − 23.6)	− 46.4 (− 51.4 to − 40.4)	− 42.1 (− 47.5 to − 35.5)	− 40.6 (− 46.1 to − 33.7)	− 49.5 (− 54.5 to − 43.4)
Gwangju	− 59.2 (− 63.9 to − 53.1)	− 56.8 (− 61.3 to − 51)	− 73.5 (− 76.4 to − 69.8)	− 38.9 (− 45.5 to − 30.4)	− 62.7 (− 67 to − 57)
Daejeon	− 29.8 (− 38.3 to − 18.7)	− 34 (− 41.4 to − 24.6)	− 38.9 (− 46 to − 29.8)	− 24.1 (− 32.7 to − 12.9)	− 45.3 (− 52 to − 36.3)
Ulsan	− 47.1 (− 53 to − 39.7)	− 41.6 (− 47.6 to − 34.1)	− 38 (− 44.7 to − 29.4)	− 24.7 (− 32.9 to − 14.1)	− 37.8 (− 44.7 to − 29)
Sejong	52 (20.5 to 105.7)	− 19.9 (− 35.4 to 5.5)	− 19.6 (− 35.6 to 6.8)	− 28.7 (− 42.8 to − 5.4)	− 24.6 (− 40.7 to 3.4)
Gyeonggi	− 35.3 (− 40.4 to − 29.1)	− 47.7 (− 51.5 to − 43.3)	− 46.8 (− 50.7 to − 42.2)	− 45.1 (− 49.2 to − 40.4)	− 48.8 (− 52.8 to − 43.9)
Gangwon	− 47.4 (− 54.1 to − 38.5)	− 38 (− 45.1 to − 28.8)	− 38.3 (− 45.5 to − 28.9)	− 36.2 (− 43.6 to − 26.5)	− 52.3 (− 58.4 to − 44.1)
Chungbuk	− 52.3 (− 57 to − 46.6)	− 52.1 (− 56.5 to − 46.8)	− 44 (− 49.3 to − 37.6)	− 32.1 (− 38.4 to − 24.3)	− 52.5 (− 57.2 to − 46.5)
Chungnam	− 37.5 (− 42.7 to − 31.3)	− 42.4 (− 46.8 to − 37.3)	− 41.6 (− 46.1 to − 36.2)	− 47.1 (− 51.2 to − 42.2)	− 60 (− 63.3 to − 56.1)
Jeonbuk	− 44 (− 49.4 to − 37.2)	− 51.3 (− 55.6 to − 46.1)	− 59.6 (− 63.3 to − 55.1)	− 40.8 (− 46.1 to − 34.4)	− 44.5 (− 49.9 to − 37.8)
Jeonnam	− 9 (− 17.3 to 1.2)	− 34.2 (− 39.5 to − 27.7)	− 38.7 (− 43.8 to − 32.4)	− 33.1 (− 38.7 to − 26.4)	− 22.4 (− 29.3 to − 14.1)
Gyeongbuk	− 12.5 (− 19.9 to − 3.5)	− 29.5 (− 35.1 to − 22.9)	− 24.9 (− 31.1 to − 17.4)	− 24.4 (− 30.6 to − 16.9)	− 21.2 (− 28.1 to − 12.8)
Gyeongnam	− 69.4 (− 71.9 to − 66.4)	− 51.6 (− 55.2 to − 47.3)	− 51.4 (− 55.3 to − 46.9)	− 28.1 (− 33.7 to − 21.5)	− 58.4 (− 61.8 to − 54.3)
Jeju	− 45.8 (− 52.6 to − 36.7)	− 71 (− 74.4 to − 66.7)	− 55.2 (− 60.4 to − 48.5)	− 65.9 (− 69.9 to − 60.6)	− 69.3 (− 73 to − 64.4)

In Korea, a constant decrease in the number of patients with heat-related illnesses was observed throughout the summer (May (− 44.5%), June (− 48.7%), July (− 48.6%), August (− 40.7%), and September (− 51%)). Furthermore, in August, the second wave of COVID-19 surged in Korea, and the highest maximum temperature was observed. Moreover, the number of patients with heat-related illnesses differed by region and month. Seoul, which had the highest incidence of COVID-19 confirmed cases, constantly experienced a deficit of heat-related illness patients during the summer (May (− 56%), June (− 58.3%), July (− 58.6%), August (− 59.2%), and September (− 63.3%)). However, in Daegu, constant deficits in heat-related illness patients were observed from May to July, whereas 35.9% of the significant excesses in heat-related illness patients were observed in August. In August, Daegu experienced the highest maximum temperature of 36.1 °C and the average maximum temperature in August was 32.1 °C. In the Gyeongnam area, which was relatively less affected by COVID-19 in Korea during the summer, there was a constant decrease in the number of heat-related illness patients, with the largest decrease in May (− 69.4%) and the smallest decrease in August (− 28.1%).

**Figure 3:**
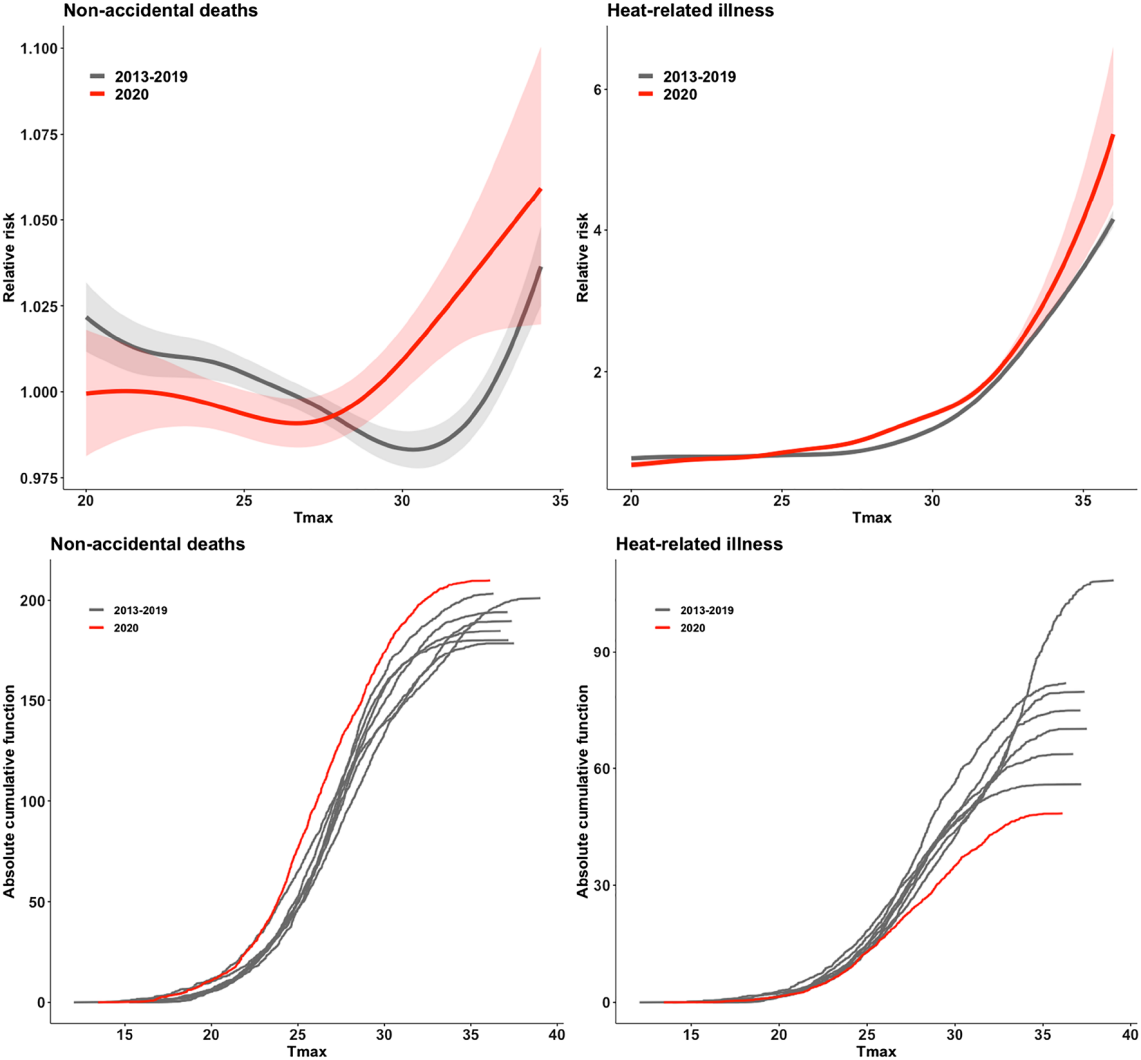
The relative risk (RR) and absolute cumulative density function (CDF) of non-accidental deaths and heat-related illness during 2013–2019 and 2020 in Korea.

The RR of non-accidental deaths and heat-related illnesses in 2020 was compared to previous years (2013–2019) (Fig. 3). The RR of non-accidental deaths at 33.1 °C was 1.00 (CI 0.99–1.01) and 1.04 (CI 1.02–1.07) in 2013–2019 and 2020, respectively. The threshold temperature (RR > 1) was lower in 2020 (29.1 °C) when compared to the value of historical years, 2013–2019 (32.8 °C). The RR of heat-related illness at 33.1 °C was 1.44 (CI 1.42–1.45) and 1.59 (CI 1.54–1.64) in 2013–2019 and 2020, respectively. In terms of the RR of heat-related illness, the threshold temperature (RR > 1) was higher in 2013–2019 (28.9 °C) than 2020 (27.4 °C). The absolute cumulative functions (ACF) of non-accidental deaths and heat-related illnesses in 2020 were compared between 2013 and 2019 (Fig. 3). The ACF of non-accidental deaths in 2020 had increased steeply compared to other historical years, and the ACF in 2020 showed the biggest value at temperatures above 25 °C. However, the ACF of heat-related illnesses was the smallest in 2020 compared to previous years. For example, the ACF of heat-related illnesses was approximately 92.14 per 100,000 people in 2018, the hottest summer, compared to 48.33 per 100,000 people in 2020.

**Figure 4:**
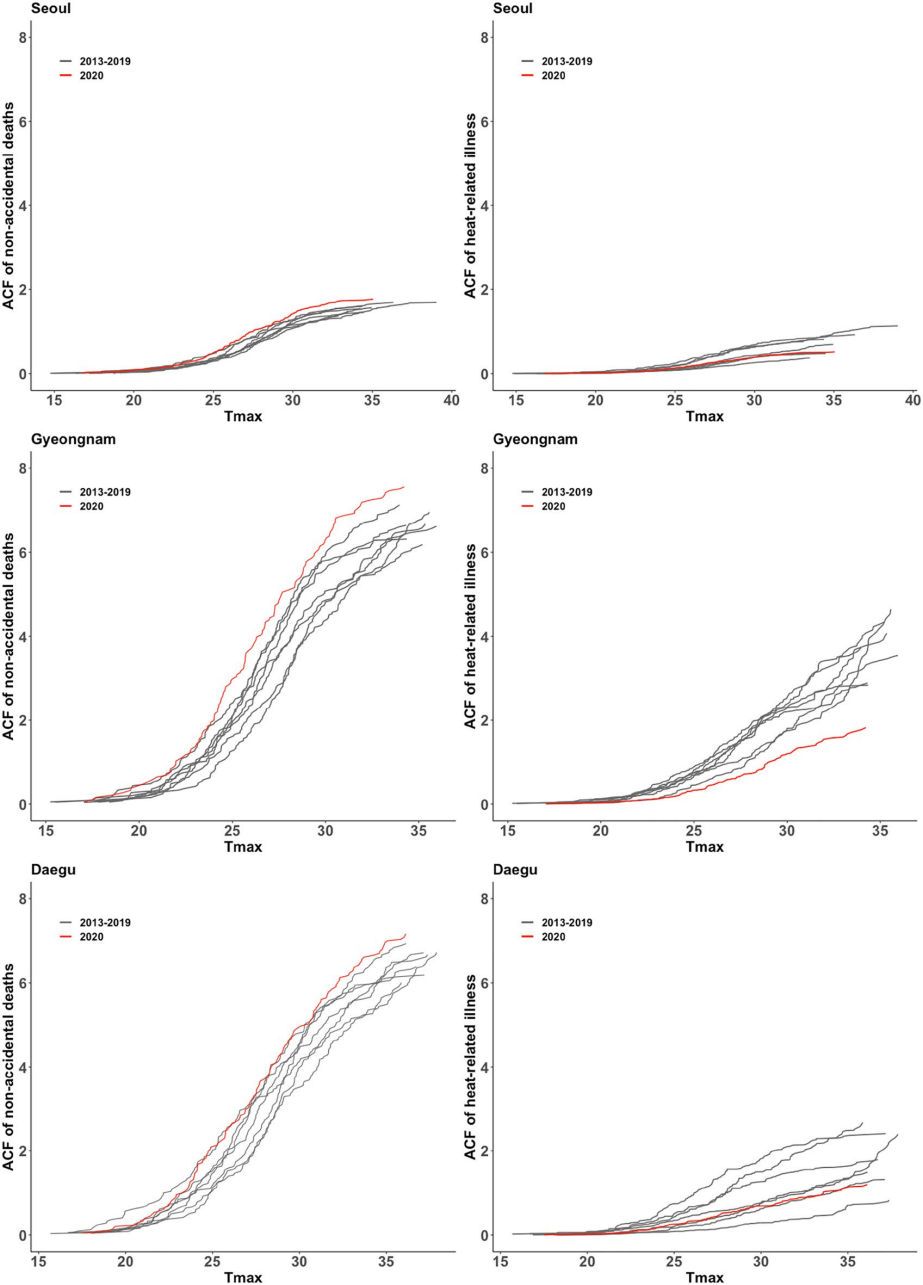
The absolute cumulative function (ACF) of non-accidental deaths and heat-related illness during 2013–2019 and 2020 in Seoul, Gyeongnam, and Daegu.

Figure 4 shows the ACF results for non-accidental deaths and heat-related illnesses in Seoul, Gyeongnam, and Daegu. In Seoul, the ACF of non-accidental deaths in 2020 was 1.43 per 100,000 people (30.06 °C), which was higher than 2018 at a similar temperature (1.17 per 100,000 people at 30.35 °C). However, the ACF for heat-related illness in 2020 was 0.52 per 100,000 people (35.05 °C), which was a 47% decrease from the ACF of 2018 at a similar temperature (0.98 per 100,000 people at 35.28 °C). In Gyeongnam, the ACF of non-accidental deaths in 2020 was 6.35 per 100,000 people (30.05 °C), which was higher than 2018 (4.86 per 100,000 people at 30.04 °C). However, the ACF of heat-related illness in 2020 was 1.82 per 100,000 people at the highest maximum temperature (34.23 °C), which was 50% less than the ACF of 2018 at a similar temperature (3.58 per 100,000 people at 34.24 °C). In Daegu, the ACF of non-accidental deaths in 2020 was 7 per 100,000 people (30.05 °C), which was higher than 2018 (5.49 per 100,000 people at 30.05 °C). The ACF of heat-related illness in 2020 was 1.14 per 100,000 people at 35.05 °C, which was a 15% decrease from the ACF of 2018 at a similar temperature (1.33 per 100,000 people at 35.05 °C).

## Discussion

During the summer of the 2020, Korea experienced 0.8% of non-accidental excess deaths, with the highest rate occurring in August. However, the number of heat-related patients decreased. The patterns of non-accidental deaths and heat-related illnesses varied by region. Particularly in Daegu, patients with excessive heat-related illnesses were observed in August. Daegu has been known as the hottest city in Korea, and its maximum temperature in August 2020 was increased to 36.1 °C. In 2020, the RR of non-accidental deaths and heat-related illnesses was higher compared to previous years. The trends in the absolute cumulative number of non-accidental deaths and heat-related illnesses were similar in the three regions (Seoul, Gyeongnam, and Daegu), indicating increased non-accidental deaths and decreased heat-related illnesses at similar temperatures in 2020.

An unprecedented number of patients with heat-related illnesses were reported in the 2018 heatwave, during the hottest summer in Korea. Abnormally hot temperatures have been recorded due to the strong and northwestward-extended North Pacific high, which led to an extraordinary heatwave in Korea^31^. During the heatwave in 2018, 2.7-fold more heat-related illnesses and 2.2–6.9-fold more deaths were reported compared to previous seven years. The impact of heatwaves is more severe in rural areas^32^. In Korea, the acute health effects of heatwaves on older people living in rural areas were higher, and their vital signs showed an immediate increase in indoor temperature as they could not afford air-conditioning^33^.

Excess non-accidental deaths increased by 0.8% in Korea, with 3% of the highest increase in August during the pandemic and approximately 4% of deaths being directly related to COVID-19. Italy (February–May, 30%; throughout 2020: 16%)^34,35^, Spain (January–September, 12%)^36^, France (January–September, 3.3%)^37^, and the United States (throughout 2020: 12%)^38^ reported excess deaths in 2020. In Portugal, which experienced a severe heatwave in 2020, more than half of the heat-related deaths increased during the summer of 2020^23^. Korea had fewer excess deaths than other countries; however, pandemic-focused healthcare systems may have had some impact on non-accidental deaths. The Korean government operated public health emergency level 4 on February 23, 2020 and prioritized most emergency units and hospital rooms for COVID-19 patients. During the first five months of the pandemic, restrictions on tuberculosis treatment were reported in Korea since many pulmonologists had been put on the frontlines^39^. Women, urban dwellers, and individuals without chronic diseases experience delays in health screening and non-urgent medical visits^40^. Such changes in the healthcare system may have led to excess deaths during the summer in Korea.

The number of patients with heat-related illnesses in Korea decreased during the study period, except in Daegu. During COVID-19, due to the fear of viral infection and the different usage of healthcare resources in healthcare facilities, changes in healthcare-seeking behavior were reported^41,42^. In Korea, social distancing was implemented at the beginning of the pandemic, and timely changes in such interventions were made considering the incidence of COVID-19 confirmed cases^43^. In September, as the Korean government implemented the level 2.5 and level 2 social distancing measures, the number of patients with heat-related illnesses decreased by approximately half. Similarly, in Japan, reduction in ambulance transport due to heat-related illnesses have reported as well^44^.

In Daegu, the number of patients with heat-related illnesses increased in August, with a high maximum temperature compared to the previous seven years. Daegu was the only city that exhibited a significant increase in the number of patients with heat-related illnesses in August. During the summer of 2020, 11 days of heatwaves were experienced in Daegu from August 11 to 21, and the incidence of COVID-19 confirmed cases was comparably smaller than the epicenter area. This situation may have enabled patients with heat-related illnesses to visit hospitals with relaxed social distancing measures in the Daegu area, known as Distancing in Life. As the level of social distancing changed to level 2 on August 22, Daegu showed a similar number of heat-related illness patients as predicted in September without severe temperature exposure. This result suggests that the concurrent existence of a severe heatwave and pandemic, coupled with the implementation of non-pharmaceutical measures, is highly likely to impact changes in healthcare-seeking behaviors.

During the pandemic, the RR of non-accidental deaths and heat-related illnesses was higher than that of previous years. Such overlapping risks of COVID-19 and heatwaves could expose high-risk groups of heat-related illness to the new risk of COVID-19, worsening health conditions^45^. Based on our findings, the total number of patients with heat-related illnesses decreased in 2020 compared to previous years, and Gyeongnam showed the highest ACF of heat-related illnesses in 2020. The residents of Gyeongnam, influenced by the low incidence of confirmed COVID-19 cases, may have been more able to seek healthcare services, as suggested by a previous study on changes in healthcare-seeking behaviors in highly contaminated areas^46^. In highly affected areas, the avoidance of healthcare has been reported in specific population^46^. Such changes in health-seeking behaviors can lead to delays in treatment and worsen health outcomes.

Our study has scope for further improvement. First, the nonlinear and delayed effects of temperature on health must be considered. The data used in the study were limited to the summer period, and the focus of the modeling approach was to estimate the baseline of non-accidental deaths and heat-related illnesses. Second, owing to the coinciding events of COVID-19 and heatwaves in the summer of 2020, it was challenging to differentiate the specific health impacts caused by each risk factor. It should be noted that the observed impact might have been more severe if an intense heatwave had occurred during the pandemic, considering that Korea experienced a relatively mild number of heatwave days during the first year of the pandemic. Third, careful interpretation of the relative risk results is needed, as we compared the data from the historical five years (2013–2019) with those from 2020. Notably, the findings derived from 2020 solely reflect the temperature characteristics observed during that specific period. Finally, as this study only examined the health impacts in 2020, it is important to investigate the long-term effects of the pandemic in relation to the intensity of heatwaves each year.

However, this study comprehensively examined the changes in both mortality and morbidity during the summer of the first pandemic year. This approach sheds light on the potential adverse health risks that arise when a heatwave and pandemic occur simultaneously. This implies that healthcare policies need to be carefully designed based on relative risk analysis and total health impacts, not just pandemics or heatwaves alone. In the future, a new pandemic and more severe heatwaves may emerge. A multifunctional healthcare policy and a risk-minimizing healthcare system are urgently required.

## Conclusion

In conclusion, our findings indicate that there are regional variations in non-accidental deaths and heat-related illnesses during the summer months, with a notable decrease in the number of heat-related illness patients, except in Daegu in August. These results emphasize the importance of considering the healthcare-seeking behaviors of individuals affected by heat-related illnesses, especially when facing simultaneous risks such as heatwaves, in the implementation of effective countermeasures during the pandemic.

### Supplementary Information

Supplementary Information.

## Data Availability

The datasets used and analysed during the current study available from the corresponding author on reasonable request. Mortality datasets generated and analyzed during the study are publicly available from MDIS services. https://mdis.kostat.go.kr/dwnlSvc/ofrSurvSearch.do?curMenuNo=UI_POR_P9240. Heat-related illness datasets were downloaded from the National Health Insurance Service using a customized DB, https://nhiss.nhis.or.kr/bd/ab/bdaba021eng.do. Regional population data were downloaded from the Ministry of the Interior and Safety website, https://jumin.mois.go.kr/#. Regional maximum temperature data were obtained from a study conducted by Yang et al.^26^, https://www.kci.go.kr/kciportal/landing/article.kci?arti_id=ART002552345.
