# Supplementary material for: Analysis of compound health impacts of heatwave and COVID-19 in Korea from May to September in 2020
Source: Sci Rep. 2023 Sep 9;13:14880. doi: 10.1038/s41598-023-41880-1 (PMC10492780; doi:10.1038/s41598-023-41880-1)
Supplement: Supplementary file 1 — Supplementary Information. [file 41598_2023_41880_MOESM1_ESM.docx]

**Supplementary materials**

**Title.** Analysis of Compound Health Impacts of Heatwave and COVID-19 in Korea from May to September in 2020

**Authors.** Haemin Park^1^, Sang-Min Lee^1^, Woo Joong Kim ^1^, Yeora Chae^1^*

**Affiliations.**

^1^*Korea Environment Institute, 370 Sicheong-daero, Sejong 30147, Republic of Korea*

^*^**Correspondence****.**

E-mail addresses: [yrchae@kei.re.kr](mailto:yrchae@kei.re.kr) (Y. C.)

**Table S1.** The validity results of non-accidental deaths by using different temperature sources.

|  | Averaged Tmax spatially gridded data | | Population adjusted averaged  Tmax from spatially gridded data | | Average Tmax from  KMA | |
| --- | --- | --- | --- | --- | --- | --- |
| Region | MAE | RMSE | MAE | RMSE | MAE | RMSE |
| Seoul | 1.45 | 10.77 | 1.45 | 10.77 | 1.46 | 10.76 |
| Busan | 1.03 | 8.08 | 1.03 | 8.08 | 1.13 | 8.09 |
| Daegu | 0.57 | 6.25 | 0.57 | 6.25 | 0.58 | 6.25 |
| Incheon | -0.04 | 5.42 | -0.04 | 5.42 | -0.03 | 5.42 |
| Gwangju | 0.94 | 3.98 | 0.94 | 3.98 | 0.94 | 3.98 |
| Daejeon | -0.81 | 4.41 | -0.81 | 4.41 | -0.83 | 4.41 |
| Ulsan | -0.88 | 3.63 | -0.88 | 3.63 | -0.88 | 3.63 |
| Sejong | -0.04 | 1.70 | -0.04 | 1.70 | -0.05 | 1.70 |
| Gyeonggi | -0.22 | 13.72 | -0.21 | 13.72 | -0.22 | 13.72 |
| Gangwon | 0.13 | 5.67 | 0.12 | 5.67 | 0.10 | 5.67 |
| Chungbuk | -1.23 | 4.75 | -1.25 | 4.76 | -1.29 | 4.76 |
| Chungnam | 0.05 | 6.22 | 0.05 | 6.22 | 0.05 | 6.22 |
| Jeonbuk | -0.27 | 6.06 | -0.26 | 6.06 | -0.29 | 6.06 |
| Jeonnam | 0.52 | 6.84 | 0.51 | 6.84 | 0.50 | 6.84 |
| Gyeongbuk | 0.23 | 6.96 | 0.24 | 6.96 | 0.21 | 6.96 |
| Gyeongnam | -0.03 | 7.83 | -0.05 | 7.83 | -0.07 | 7.83 |
| Jeju | -0.27 | 2.91 | -0.27 | 2.91 | -0.26 | 2.91 |
| Average | 0.07 | 6.19 | 0.06 | 6.19 | 0.06 | 6.19 |

**Figure S1.** The mean value of non-accidental deaths mortality, heat-related illness occurrence, and maximum temperature during May-Sep in Korea by region (2013-2020).


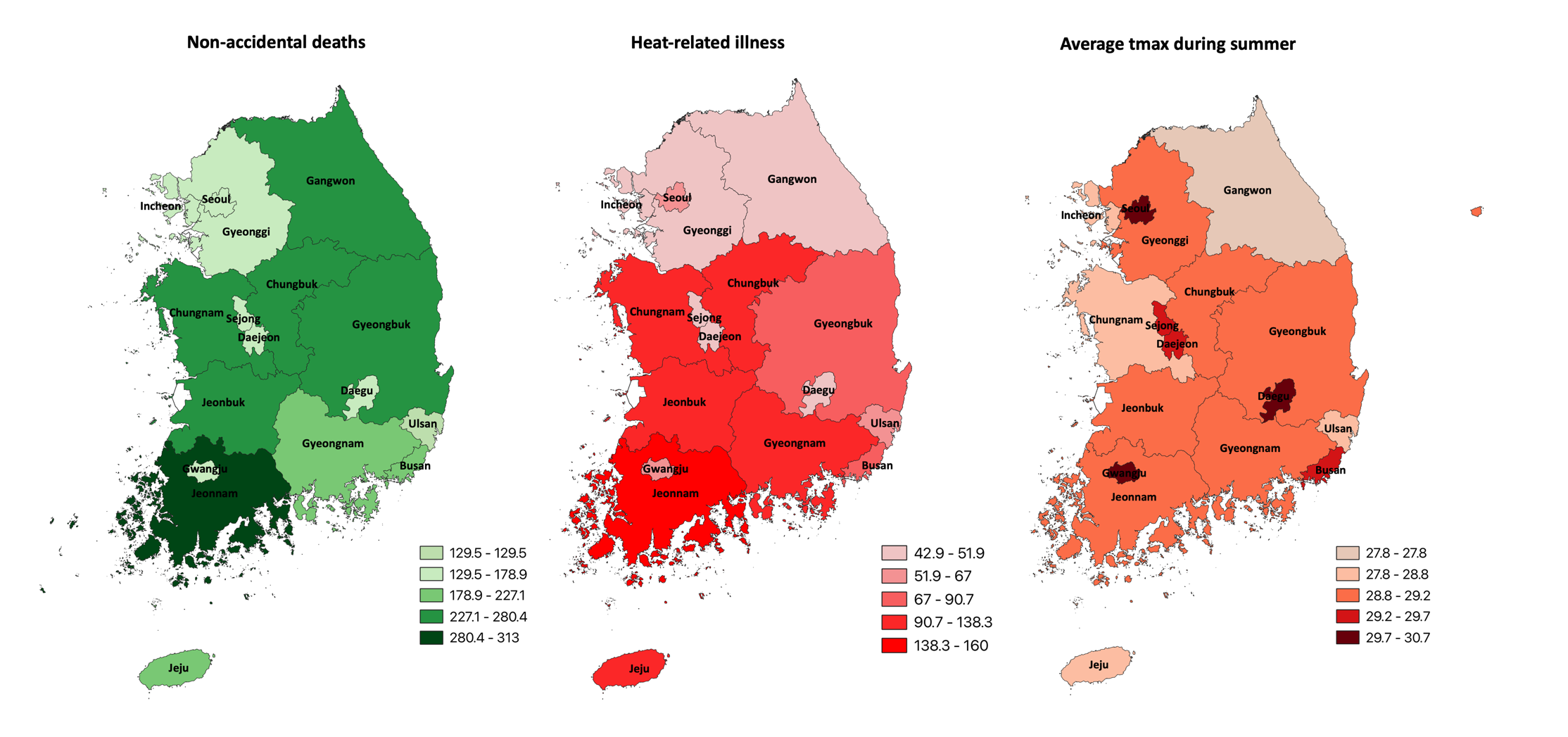


**Table S2.** The non-accidental mortality by region during May-September in 2013-2020.

| Region | 2013 | 2014 | 2015 | 2016 | 2017 | 2018 | 2019 | 2020 |
| --- | --- | --- | --- | --- | --- | --- | --- | --- |
| Seoul | 146.6 | 146.9 | 154.9 | 156.2 | 157.0 | 165.3 | 164.5 | 170.9 |
| Busan | 192.4 | 194.4 | 201.5 | 207.4 | 212.2 | 226.7 | 224.6 | 236.9 |
| Daegu | 161.3 | 165.6 | 170.9 | 180.2 | 182.4 | 182.2 | 186.8 | 191.8 |
| Incheon | 155.2 | 159.6 | 155.8 | 166.6 | 166.7 | 181.3 | 181.2 | 188.1 |
| Gwangju | 156.5 | 169.3 | 176.5 | 173.4 | 190.4 | 183.4 | 185.1 | 196.6 |
| Daejeon | 154.2 | 146.3 | 157.5 | 159.9 | 163.2 | 176.4 | 183.1 | 187.5 |
| Ulsan | 123.7 | 124.1 | 127.9 | 123.0 | 120.9 | 134.2 | 138.9 | 143.2 |
| Sejong | 220.2 | 174.2 | 160.8 | 146.1 | 136.7 | 142.0 | 123.9 | 140.2 |
| Gyeonggi | 143.8 | 143.4 | 145.8 | 153.2 | 157.0 | 163.2 | 165.9 | 169.0 |
| Gangwon | 239.8 | 247.3 | 256.5 | 257.0 | 264.3 | 275.2 | 277.9 | 287.1 |
| Chungbuk | 252.1 | 225.0 | 237.3 | 241.9 | 240.5 | 244.6 | 253.8 | 258.7 |
| Chungnam | 229.7 | 234.4 | 240.5 | 247.3 | 257.1 | 255.6 | 263.8 | 269.8 |
| Jeonbuk | 247.3 | 250.3 | 250.6 | 263.3 | 272.9 | 283.0 | 289.7 | 297.0 |
| Jeonnam | 294.7 | 296.3 | 292.9 | 303.7 | 328.1 | 323.3 | 327.3 | 337.9 |
| Gyeongbuk | 255.8 | 268.7 | 268.2 | 271.3 | 283.6 | 291.8 | 294.3 | 309.5 |
| Gyeongnam | 206.1 | 211.6 | 223.6 | 225.1 | 223.8 | 234.6 | 239.6 | 252.4 |
| Jeju | 184.9 | 181.3 | 174.6 | 188.6 | 200.6 | 199.8 | 208.6 | 196.0 |

**Table S3.** The occurrence of heat-related illness by region during May-September in 2013-2020.

| Region | 2013 | 2014 | 2015 | 2016 | 2017 | 2018 | 2019 | 2020 |
| --- | --- | --- | --- | --- | --- | --- | --- | --- |
| Seoul | 37.6 | 48.2 | 50.2 | 69.5 | 80.1 | 110.6 | 90.0 | 49.9 |
| Busan | 108.3 | 82.8 | 100.5 | 108.3 | 82.5 | 107.9 | 86.2 | 48.9 |
| Daegu | 71.8 | 64.4 | 48.0 | 22.3 | 35.8 | 64.8 | 40.2 | 32.1 |
| Incheon | 52.5 | 37.2 | 34.9 | 43.4 | 43.0 | 81.6 | 59.5 | 34.7 |
| Gwangju | 65.3 | 48.2 | 56.9 | 68.9 | 58.4 | 95.2 | 67.6 | 29.5 |
| Daejeon | 56.5 | 39.4 | 41.8 | 47.0 | 41.5 | 71.1 | 49.9 | 30.8 |
| Ulsan | 46.0 | 41.1 | 48.4 | 61.9 | 58.6 | 94.7 | 56.5 | 49.6 |
| Sejong | 67.9 | 57.0 | 57.4 | 45.3 | 49.3 | 60.8 | 44.6 | 32.9 |
| Gyeonggi | 37.8 | 29.1 | 40.3 | 52.6 | 42.3 | 65.3 | 47.3 | 28.2 |
| Gangwon | 36.5 | 31.9 | 41.2 | 48.5 | 51.2 | 88.4 | 45.2 | 34.2 |
| Chungbuk | 110.8 | 102.7 | 125.3 | 126.3 | 112.7 | 137.2 | 82.1 | 51.6 |
| Chungnam | 133.7 | 110.8 | 133.8 | 141.8 | 123.6 | 169.2 | 150.7 | 77.0 |
| Jeonbuk | 114.2 | 72.1 | 79.2 | 140.0 | 150.7 | 206.4 | 108.8 | 72.1 |
| Jeonnam | 177.5 | 114.0 | 133.9 | 172.9 | 148.7 | 217.1 | 198.7 | 117.0 |
| Gyeongbuk | 75.7 | 52.2 | 70.9 | 90.8 | 82.7 | 127.9 | 104.1 | 84.0 |
| Gyeongnam | 144.5 | 95.2 | 97.3 | 137.6 | 119.8 | 156.8 | 125.6 | 60.7 |
| Jeju | 86.2 | 41.0 | 37.3 | 135.0 | 180.0 | 277.0 | 228.2 | 122.0 |

**Table S4.** The mean value of maximum temperature during May-September in 2013-2020.

| Region | 2013 | 2014 | 2015 | 2016 | 2017 | 2018 | 2019 | 2020 |
| --- | --- | --- | --- | --- | --- | --- | --- | --- |
| Seoul | 30.1 | 30.1 | 30.8 | 31.3 | 30.0 | 31.0 | 30.5 | 29.4 |
| Busan | 30.1 | 28.4 | 28.5 | 29.7 | 30.5 | 30.1 | 29.3 | 29.0 |
| Daegu | 31.6 | 29.8 | 30.2 | 31.1 | 31.6 | 31.3 | 30.3 | 29.7 |
| Incheon | 28.4 | 28.8 | 29.0 | 29.0 | 28.5 | 29.3 | 28.8 | 27.7 |
| Gwangju | 31.2 | 29.9 | 30.2 | 30.9 | 31.1 | 31.6 | 30.2 | 29.0 |
| Daejeon | 30.1 | 29.0 | 29.6 | 30.4 | 29.7 | 30.6 | 29.6 | 28.9 |
| Ulsan | 30.6 | 28.3 | 28.2 | 28.6 | 29.4 | 28.9 | 28.2 | 28.3 |
| Sejong | 29.5 | 28.7 | 29.6 | 30.2 | 29.7 | 30.4 | 29.6 | 28.8 |
| Gyeonggi | 28.7 | 28.6 | 29.3 | 29.6 | 28.7 | 29.5 | 28.8 | 27.9 |
| Gangwon | 27.9 | 27.5 | 28.0 | 28.1 | 27.7 | 28.5 | 28.0 | 27.0 |
| Chungbuk | 29.1 | 28.4 | 29.2 | 29.7 | 29.1 | 29.9 | 29.1 | 28.1 |
| Chungnam | 28.9 | 28.1 | 28.6 | 29.4 | 28.8 | 29.6 | 28.6 | 27.8 |
| Jeonbuk | 29.5 | 28.1 | 28.8 | 29.8 | 29.3 | 30.0 | 28.7 | 28.0 |
| Jeonnam | 29.7 | 27.9 | 28.6 | 29.5 | 29.7 | 30.0 | 28.6 | 28.1 |
| Gyeongbuk | 29.8 | 28.3 | 28.9 | 29.5 | 29.6 | 29.6 | 28.9 | 28.1 |
| Gyeongnam | 30.1 | 28.2 | 28.5 | 29.5 | 30.1 | 29.7 | 29.0 | 28.4 |
| Jeju | 29.3 | 27.4 | 27.6 | 28.8 | 29.4 | 28.7 | 28.0 | 27.9 |

**Table S5.** The mean value of maximum temperature during May-September in 2013-2020.

| Region | 2013 | 2014 | 2015 | 2016 | 2017 | 2018 | 2019 | 2020 |
| --- | --- | --- | --- | --- | --- | --- | --- | --- |
| Seoul | 33.5 | 30.4 | 32.8 | 34.6 | 30.7 | 34.5 | 33.4 | 31.4 |
| Busan | 34.2 | 28.7 | 31.4 | 33.3 | 32.5 | 33.3 | 32.1 | 33.1 |
| Daegu | 34.9 | 29.3 | 32.3 | 35.2 | 32.7 | 34.7 | 33.0 | 33.6 |
| Incheon | 31.7 | 29.6 | 30.7 | 32.4 | 30.0 | 33.4 | 31.9 | 29.9 |
| Gwangju | 34.6 | 29.4 | 32.0 | 34.6 | 32.9 | 35.2 | 33.5 | 32.8 |
| Daejeon | 33.4 | 28.6 | 31.5 | 33.8 | 30.9 | 34.2 | 32.6 | 32.4 |
| Ulsan | 34.7 | 28.6 | 31.2 | 31.8 | 31.1 | 32.1 | 30.8 | 32.6 |
| Sejong | 32.6 | 28.3 | 31.4 | 33.5 | 30.4 | 34.1 | 32.5 | 31.9 |
| Gyeonggi | 31.8 | 28.8 | 31.0 | 32.5 | 29.5 | 33.1 | 31.5 | 30.1 |
| Gangwon | 31.3 | 27.3 | 30.0 | 31.0 | 28.3 | 31.5 | 30.5 | 29.7 |
| Chungbuk | 32.1 | 27.9 | 31.0 | 32.9 | 29.9 | 33.4 | 31.7 | 31.3 |
| Chungnam | 32.2 | 28.0 | 30.7 | 32.6 | 30.3 | 33.3 | 31.6 | 30.9 |
| Jeonbuk | 32.6 | 28.0 | 30.8 | 33.0 | 30.8 | 33.3 | 31.6 | 31.7 |
| Jeonnam | 33.3 | 28.3 | 30.8 | 33.3 | 31.8 | 33.3 | 31.5 | 31.9 |
| Gyeongbuk | 33.3 | 27.9 | 31.2 | 33.1 | 30.5 | 32.9 | 31.5 | 31.9 |
| Gyeongnam | 33.7 | 28.3 | 31.0 | 33.3 | 32.0 | 32.9 | 31.8 | 32.1 |
| Jeju | 32.7 | 28.3 | 30.1 | 32.4 | 32.3 | 32.1 | 31.1 | 32.2 |

**Table S6.** The mean value of maximum temperature in August, 2013-2020.

| Region | 2013 | 2014 | 2015 | 2016 | 2017 | 2018 | 2019 | 2020 |
| --- | --- | --- | --- | --- | --- | --- | --- | --- |
| Seoul | 36.6 | 36.1 | 37.1 | 38.9 | 37.2 | 41.1 | 38.2 | 36.7 |
| Busan | 38.8 | 34.0 | 36.7 | 38.2 | 37.9 | 39.1 | 36.2 | 37.6 |
| Daegu | 38.3 | 35.4 | 38.7 | 39.5 | 38.4 | 39.5 | 37.5 | 37.0 |
| Incheon | 34.5 | 36.4 | 34.7 | 35.3 | 35.9 | 38.2 | 36.4 | 35.1 |
| Gwangju | 37.7 | 33.8 | 37.2 | 38.4 | 38.1 | 40.1 | 37.3 | 35.7 |
| Daejeon | 36.2 | 32.8 | 36.8 | 37.6 | 36.9 | 39.6 | 36.9 | 36.0 |
| Ulsan | 40.0 | 34.3 | 38.2 | 36.9 | 36.6 | 37.5 | 35.5 | 36.1 |
| Sejong | 35.2 | 33.1 | 36.2 | 36.9 | 35.8 | 39.4 | 36.9 | 36.3 |
| Gyeonggi | 33.8 | 34.9 | 35.5 | 35.6 | 36.0 | 38.8 | 36.1 | 34.7 |
| Gangwon | 33.8 | 33.9 | 34.0 | 35.3 | 33.9 | 37.2 | 34.3 | 34.1 |
| Chungbuk | 34.3 | 33.3 | 36.2 | 36.1 | 36.2 | 38.8 | 36.3 | 35.3 |
| Chungnam | 34.6 | 32.5 | 35.4 | 35.6 | 36.1 | 38.0 | 35.7 | 35.0 |
| Jeonbuk | 35.5 | 31.9 | 36.1 | 36.0 | 36.2 | 37.9 | 35.4 | 34.3 |
| Jeonnam | 36.2 | 32.3 | 35.8 | 36.7 | 36.6 | 37.1 | 34.8 | 34.5 |
| Gyeongbuk | 36.3 | 34.1 | 37.1 | 37.3 | 36.2 | 38.4 | 36.0 | 35.1 |
| Gyeongnam | 37.3 | 32.8 | 36.0 | 37.2 | 37.5 | 37.1 | 35.7 | 36.0 |
| Jeju | 36.0 | 31.7 | 34.1 | 34.2 | 35.1 | 35.1 | 33.9 | 34.3 |

**Table S7.** The characteristics of maximum temperature and COVID-19 confirmed cases by region in Korea.

| **Region** | **Average Tmax** | **Average Tmax in Aug** | **Maximum Tmax in Aug** | **COVID-19 confirmed cases during May-Sep (per 100,000)** | **Cumulative COVID-19 confirmed cases (per 100,000)** |
| --- | --- | --- | --- | --- | --- |
| **Seoul** | 26.9 | 29.6 | 34.7 | 48.2 | 54.7 |
| **Busan** | 25.7 | 30.5 | 33.7 | 8.0 | 11.8 |
| **Daegu** | 27.8 | 32.1 | 36.1 | 10.5 | 266.3 |
| **Incheon** | 25.5 | 28.6 | 33.4 | 27.1 | 30.2 |
| **Gwangju** | 27.2 | 31.6 | 34.3 | 32.1 | 34.1 |
| **Daejeon** | 26.7 | 30.7 | 33.9 | 21.9 | 24.6 |
| **Ulsan** | 25.6 | 30.6 | 33.6 | 7.7 | 10.8 |
| **Sejong** | 26.8 | 30.4 | 34.2 | 8.4 | 21.4 |
| **Gyeonggi** | 26.4 | 29.2 | 33.7 | 27.6 | 32.6 |
| **Gangwon** | 24.5 | 27.7 | 31.8 | 11.1 | 14.5 |
| **Chungbuk** | 26.3 | 29.8 | 33.5 | 7.9 | 10.7 |
| **Chungnam** | 26.2 | 29.9 | 33.7 | 16.1 | 22.8 |
| **Jeonbuk** | 26.1 | 30.4 | 32.8 | 6.1 | 7.1 |
| **Jeonnam** | 26.0 | 30.5 | 33.1 | 8.3 | 9.1 |
| **Gyeongbuk** | 26.4 | 30.6 | 34.1 | 7.0 | 58.8 |
| **Gyeongnam** | 26.2 | 30.5 | 34.2 | 5.2 | 8.7 |
| **Jeju** | 25.2 | 30.0 | 31.9 | 6.8 | 8.7 |
